# The Treatment of Lupus Vulgaris

**Published:** 1892-09-03

**Authors:** 


### THE TREATMENT OF LUPUS VULGARIS.

It is, unfortunately, too true that modern treatment has hardly kept pace with the great advances made in the study of pathology, and in no disease is this more true than in lupus vulgaris. This is not, however, the place to discuss the various views that have been brought forward for and against the claims of the tubercle bacillus as the cause of lupus vulgaris, but even the disbelievers in the agency of that micro-organism in this disease admit that some micro-parasite is present in the tissue, though they deny that the bacillus found is the same as that present in tubercular lesions. All pathologists, however, agree that the disease is not a constitutional, but a local disorder, and hence the modern treatment is limited to local manifestation. This does not imply that the patient's general health should not be attended to, and be treated with tonics or drugs indicated by the manifest condition, but rather that no cure can be hoped for by constitutional treatment alone, for the processes of repair of the diseased tissue are much facilitated by the healthy condition of the patient.

Local treatment, on which alone we shall touch, has been attempted in numerous ways, and from the very number of methods used conclusions might be readily drawn that, even if one reagent results in a cure, as its advocate generally claims, in the hands of most physicians failure to obtain a perfectly satisfactory result is the rule.

Numerous pastes of different composition have at times been recorded as curing the disease. Thus we have a paste of chloride of zinc, one of arsenic, recommended by Hebra, and one of salicylic acid, by Marshall. They are all useful as escharotics, but their application is attended with much pain, and the extent of their action is at times hard to limit. The pain may be relieved by a 20 per cent. solution of cocaine previously applied. More strong caustics are at times very useful: such, for example, as fuming nitric acid, and acid nitrate of mercury, while the electro cautery and Paquelin's cautery may be used to destroy small nodules of growth, but the former two have the objection of penetrating deeply into the tissue and producing in many cases more destruction than was in tended, while the burnt tissue formed by the latter two prevents the observer seeing whether the diseased parts are wholly destroyed. The cautery has one great advantage, which is that the hæmorrhage, always free from the vascular lupus tissue, is arrested by the hot wire.

By far the most satisfactory results are obtained by removal of the lupus growth by the sharp spoon or by a scarifier, the former being the instrument most in favour. Volkman's sharp spoon is too well known to require any description, but its use in lupus is comparatively recent, and a few details will not be amiss. The most convenient size is certainly a small oval-shaped one rather than the circular form, and the edge should be fairly sharp, without being too much so. An anæsthetic is required during its use, as the pain is considerable, but if the patch of growth is small, laughing gas will be found to be quite sufficient. The anæsthetist must make his own choice if the operation is to be more extensive, but he will find that in giving chloroform he will avoid getting the mouthpiece of the inhaler covered with blood and removed tissue, and he will leave the face of the patient more exposed for the operator.

A very little experience will give the operator the delicacy of touch required to distinguish between the healthy skin and the lupus tissue. And he will find that with a free hand he can scrape away all the disease parts, and leave a raw surface for granulations to spring up from. One operation seldom suffices to cure lupus; again and again it has to be repeated, in many cases till both patient and operator begin to lose heart, and believe, as some have stated, that lupus is an incurable disease. But even if a cure is not obtained, a considerable amount of improvement generally takes place, and what was formerly diseased tissue is replaced by a firm though often unsightly scar. Harm seldom results from this method of treatment, the most serious being the production of keloid.

On the same principle Pick's scarifier may be used. This consists of several small knives fixed close together at the end of a handle. The tissue is lightly but firmly cut in all directions, but the serious consideration that the sense of touch cannot appreciate so well the difference between healthy and diseased tissue has led to the abandonment of this instrument for the spoon. On the completion of either scarification or scraping with the spoon, some simple antiseptic application is laid on the sore parts, the hæmorrhage, which is generally pretty free, being arrested by pressure.

We believe that at the present time the injection of Koch's fluid tuberculin has entirely fallen into disuse, if not into disrepute for the cure of lupus, though it was in this form of tubercular disease, as they claimed, the effects of the fluid were most readily observed and most markedly efficacious. It certainly did in many cases produce a most marked result, if not improvement in the local conditions, but of the many cases in which it was tried, none have, we believe, been permanently benefited, and a great many are as bad as they were before they were subjected to this process. Not that the theory of the whole thing was wrong, for if we could find some substance which, when introduced into the circulation, would act on the peculiar granulation tissue of lupus and cause its necrosis, a cure would result. Unfortunately, tuberculin seemed to stop short of complete destruction of the lupus tissue, and hence fell, not unjustifiably, into disrepute.

During the last six months, at the Bristol General Hospital, Dr. Harrison has been trying the effects of free sulphur on lupus tissue. The use of sulphur, if we remember right, is not new, but its method of application by Dr. Harrison for the cure of lupus is a new and hopeful departure, we hope, in the right direction. The method of producing sulphur in immediate contact with the deeper parts of the diseased tissue, is obtained by a very simple chemical reaction by which "nascent" sulphur is liberated by the action of dilute hydrochloric acid on hyposulphite of soda. Overnight Dr. Harrison applies to the lupus granulations a solution of hyposulphite of soda, grains 40 to ℥i, and this is allowed to soak into the tissue for twelve hours or so, evaporation being prevented by a covering of gutta-percha tissue or oilskin. In the morning a solution of hydrochloric acid (B. P.) Mv to ℥i of water is applied, when "nascent" sulphur is liberated in the innermost recesses of the diseased parts. The operation is almost painless, and could, we expect, be made absolutely so by the addition of cocaine. At the Nottingham meeting of the British Medical Association, when Dr. Harrison made his first communication on his work, several cases were read, and one was shown in which great improvement, if not a cure, had been effected by this treatment, and though some of his audience stated they saw "apple jelly" tissue, we are not without hope that permanent cicatrisation of the tissue may follow a further application of "nascent" sulphur. The most satisfactory case shown was that of a boy who had undergone treatment at more than one of our provincial hospitals without relief.

Lupus is certainly not an incurable disease, as some physicians wish us to believe; it is, everyone admits, a most disappointing one to treat, but the very fact that it has as yet baffled our attempts to destroy it should make us redouble our efforts to discover a cure for the disease which produces such hideous disfigurements on the most cherished part of a human being's body.

